# Job vacancy at the European Centre for Disease Prevention and Control

**DOI:** 10.2807/1560-7917.ES.2016.21.42.30375

**Published:** 2016-10-20

**Authors:** Eurosurveillance editorial team

**Affiliations:** 1 European Centre for Disease Prevention and Control (ECDC), Stockholm, Sweden

**Keywords:** Public health training

The European Centre for Disease Prevention and Control (ECDC) invites applications for the following position:

• Head of Section Public Health Training (PHC)

The deadline is 14 November 2016. For more information, visit the ECDC website: http://ecdc.europa.eu/en/aboutus/jobs/Pages/JobOpportunities.aspx

